# Aging as Readiness and Wariness

**DOI:** 10.1093/geroni/igab046.1829

**Published:** 2021-12-17

**Authors:** David Ekerdt

**Affiliations:** University of Kansas, Lawrence, Kansas, United States

## Abstract

Gerontology concerns itself with events in time, either things that have happened or things that may happen. In the former, our work is to describe and explain. In the latter, the occurrence of events is unknowable, but we can nonetheless study people’s imagination of them (how it arises) and how that imagination shapes behavior and attitudes in the present (how it matters). The subjective experience of aging, thus, is one of looking ever forward—welcoming, waiting for, or hoping to avoid what the future may hold. This personal experience of aging toggles between readiness and wariness of the time ahead, one stance incurring or else eclipsing the other. Transitions are fruitful opportunities to study people’s readiness and wariness toward the time ahead, for example, widowhood, the prospect of retirement, and residential relocation. This is when people are more likely to conjure, in their minds, whom they may become. Arguably, the fundamental transition that looms and occupies aging minds (and the minds of loved ones) is not death but rather the potential passage into the “fourth age” of frailty and vulnerability. This prospect hovers above all else: its occurrence increasingly likely but its timing uncertain. About this prospect, gerontology has the capacity, nay the obligation, to promote narratives about later life that shape wariness and readiness for the practical future (e.g., financial matters, bodily care, living arrangements) as well as for the emotional reception of an old age coming ever closer.

